# Factors Related to Sexual Intercourse Among Korean Middle and High School Students

**DOI:** 10.3389/fpubh.2022.924489

**Published:** 2022-07-08

**Authors:** Joohee Shim, Jihyun Baek, Seungwoo Han

**Affiliations:** ^1^College of Nursing, Yeungnam University College, Daegu, South Korea; ^2^College of Nursing, Research Institute of Nursing Science, Jeonbuk National University, Jeonju, South Korea; ^3^Department of Emergency Medical Technology, Kyungil University, Gyeongsan, South Korea

**Keywords:** adolescent, coitus, health behavior, mental health, suicide

## Abstract

**Background:**

Social interest in sexual intercourse among teenagers is increasing in Korea. This study aimed to identify factors related to sexual intercourse among adolescents.

**Methods:**

This is a secondary analysis study using data from the 2020 Korea Youth Risk Behavior Survey, with information regarding 54,948 middle school and high school students in Korea having been analyzed. Demographics, health behavior, and mental health characteristics were included. Data evaluation involved chi-square test and binary multivariable logistic regression analysis.

**Results:**

For middle school students, sexual intercourse was related to their housing type [Odds Ratio (OR) = 10.698], smoking (OR = 5.165) and drinking (OR = 2.229) experience, suicide attempt (OR = 1.920), gender (OR = 1.632), loneliness (OR = 1.501), sadness and despair (OR = 1.493), year level (OR = 1.457), happiness (OR = 0.772), sleep status (OR = 0.716), economic status (OR = 0.705). For high school students, sexual intercourse was related to their smoking experience (OR = 4.746), housing type (OR = 3.661), drinking experience (OR = 2.840), drug use (OR = 2.511), suicide attempt (OR = 2.071), year level (OR = 1.989), sadness and despair (OR = 1.506), gender (OR = 1.429), suicide plan (OR = 1.402), loneliness (OR = 1.270), academic performance (OR = 1.165), happiness (OR = 0.865), subjective health status (OR = 0.814), and economic status (OR = 0.727). However, sleep status was statistically significant for middle school students, but not for high school students. Academic performance, drug use, suicide plan, and subjective health status were statistically significant in high school students, but not in middle school students.

**Conclusion:**

A program that can directly mediate these factors must be prepared. When conducting sex education for adolescents, mental health-related factors must also be considered.

## Introduction

Adolescence is a period of biological, social, and emotional changes, as well as heightened sexual interest (1). Adolescent health behavior is especially important as it influences both present and future health (2). In addition, the first sexual experience is a crucial life milestone that marks the beginning of an individual's sexual and reproductive life (3). It is critical to develop healthy sexual interests and a sound concept of sexuality during adolescence (4).

Sexual intercourse prevalence among Korean adolescents is 4.6% (5), which is low compared with other countries (13.2%) (6). However, the prevalence of sexual intercourse among Korean adolescents is gradually increasing (4), and early sexual intercourse experiences cause several adverse outcomes, posing an important public health issue (3). Sexual intercourse throughout adolescence is linked to physical, mental, and social consequences including unwanted pregnancy (7). If an individual begins having sexual intercourse during adolescence, they are more likely to have multiple sexual partners, engage in sexual practices such as oral and anal sex, engage in prostitution, and have a high risk of acquiring sexually transmitted infections (STIs) including human immunodeficiency virus (HIV) and acquired immunodeficiency syndrome (AIDS) (3, 8). The sexual intercourse experience of adolescents was also found to be related to demographic characteristics such as year level, gender, economic status, academic performance, and housing type (7, 9–11). Adolescents are highly influenced by their friends and are sensitive to social learning, including imitation of their friends' behavior (1). Thus, sociocultural problems, such as alcohol consumption, tobacco use, drug use, and fleeing from home, are reported to be related to sexual intercourse (2, 12–15).

Given that adolescents try to compensate for feelings of loneliness through sexual activity, those who experience loneliness tend to engage in sexual intercourse before the age of 14 and are at high risk of having two or more sexual partners (11). Mental health characteristics, such as suicidal ideation, suicidal plan, suicide attempt, loneliness, anxiety, depression, stress, happiness, and subjective health status, are also known to be related to the sexual intercourse experience of adolescents (2, 7, 12, 13, 16, 17). Per previous studies, the risk of experiencing sexual intercourse among adolescents increased as adolescents experienced various psychosocial distress indicators, such as suicide planning, loneliness, anxiety, sadness, hopelessness, and being worried (10, 11). However, it is difficult to determine which mental health characteristics are closely related to sexual intercourse. Adolescents are thought to tend to escape mental health distress through sexual intercourse (10). Therefore, it is necessary to identify which factors among various mental health indicators are associated with adolescents' sexual intercourse.

Despite analysis of several factors related to adolescents' sexual intercourse experiences, few studies have used a model to investigate the relationship between demographics, health behaviors, mental health characteristics, and adolescent sexual intercourse while controlling for other variables (5). Moreover, previous studies on Korean adolescents are very limited. A previous study of Korean high school students reported that sexual intercourse was related to risk behaviors such as smoking, drinking alcohol, using drugs, and experiencing depression (12). There was a previous study that analyzed the relationship between demographics, risk behaviors, mental health characteristics, and sexual intercourse, but only female middle school students were included (7).

To promote healthy sexual behaviors, different approaches according to school level are required, due to disparities in the prevalence of sexual activity and rates of risky behaviors between middle school and high school pupils. Therefore, this study investigated factors related to sexual intercourse among Korean adolescents, according to school level, using data from the Korea Youth Risk Behavior Survey (KYRBS), a national adolescent health statistic in Korea.

## Materials and Methods

This study was a secondary data analysis using the 2020 KYRBS to identify factors associated with sexual intercourse among Korean adolescents. Our study's specific objectives were to identify factors influencing sexual intercourse prevalence, including demographics, health behaviors, and mental health. Looking at the previous papers related to sexual intercourse, it was approached from an ecological point of view (18). The ecological perspective is a theory that lays the foundation for human growth and interaction with social structures and the influence of the social environment. By using this, we can set a meaningful direction for the correct growth of young people. According to ecological theory, four major systems (individual system, micro system, intermediate system, external system) interact within these systems. This study tried to create a conceptual framework based on the ecological system theory, but the intermediate system (family, peer, teacher support) did not fit with this study. Therefore, this researcher finally constructed a conceptual framework based on previous researches including demographic characteristics (7, 9–11), health behavior characteristics (1, 2, 13), and mental health characteristics (2, 7, 12, 13, 16, 17).

### Research Materials and Participants

The definition of adolescent presented by the World Health Organization (19) is a life stage between childhood and adulthood between the ages of 10 and 19. This unique stage in human development plays an important role in individuals' future health. The target population (aged 12–18) is middle and high school students across the country, meaning middle and high school students who actually participated in the survey. This data was used for the establishment of the national health promotion plan, and was used as the basis for youth health policies and legislation. In this study, the management regulations for the use of raw data of the Korea Centers for Disease Control and Prevention were followed, and permission was obtained for data utilization. This study was conducted in 17 cities and provinces nationwide from August 03 to November 13, 2020. This was an anonymous self-report online survey with 54,948 participants from 793 schools (398 middle schools and 395 high schools). For this study's sampling process, stratification of students in this study was completed using regional groups and school levels as stratification variables. During the sampling process, the population composition ratios of 17 cities and provinces were matched according to stratification. The raw data encompassed all students selected as the sample class, however students with long-term absences, handicapped children, and students lacking literacy skills were excluded from the sample class (5). According to the data analysis guidelines of the Korea Centers for Disease Control and Prevention, the raw data was not manipulated by the researcher, and personal information was collected in an ethically anonymized and confidential manner using a unique number that could not be identified.

### Data Collection

The 2020 KYRBS is an annual online survey conducted by the Korea Centers for Disease Control and Prevention, and it is a credible survey that contributes to identifying the health behaviors of Korean youth and calculating health indicators necessary for planning and evaluating youth health promotion projects. A week before the investigation, the Korea Centers for Disease Control and Prevention sent a notice for students to the school, and only those students who agreed to the notice were conducted the study. One day before the day of the survey, the teacher in charge finally checked whether the computers in each class were working. On the day of the survey, a code number was assigned to each student to ensure the anonymity of the students. This data clearly states that parental consent is not separately implemented as this is a credible survey conducted by the Korea Centers for Disease Control and Prevention. In each school computer room, each student was assigned a computer and a seat, and the space was widely allocated to each student to maintain privacy. The questionnaire took 15–20 min for each student.

### Study Variables

#### Sexual Intercourse

For the purposes of this study, sexual intercourse is defined as a joining of the sexual organs of a male and a female, in which the erect penis of the male is inserted into the vagina of the female, usually with the ejaculation of semen into the vagina (20). Categories of dependent variables were established based on the classification presented in the raw data. In this study, sexual intercourse was selected as the dependent variable. For the question “Have you ever had sex,” items were divided into “I have never had sex” and “I have had sex.”

#### Demographic, Health Behavior, and Mental Health Characteristics

All scale items were categorized. χ^2^-test was performed with selected variables based on the raw data. In this study, there were five, seven, and eight independent variables for demographics, health behaviors, and mental health characteristics, respectively. The variables for demographic characteristics were grade level, gender, academic performance, economic status, and housing type. In the 2020 KYRBS, the adolescents were asked subjectively to choose one of five options (upper, middle upper, middle, lower middle, lower) to answer the question: “In the last 12 months, how have you been doing in your academic performance?” and “What is the economic status of your family?” Academic performance and economic status were not used objective numerical valves (e.g., top 10% of grades, income of 3 million won or less, etc.), but were based on adolescents' individual perceptions. In this study, “upper” and “middle upper” were converted to “good,” “middle” to “fair,” and “lower middle” and “lower” to “poor”.

Health behavior variables included alcohol consumption, smoking experience, quality of sleep, drug use, prior sex education, use of contraceptives, and contraceptive methods used. In the 2020 KYRBS, the participants were asked to choose “yes” or “no” to answer for the questions: “Have you ever experienced smoking, alcohol, or drugs?”, “Have you ever experienced sex education in the last 12 months.” Sleep state was evaluated using the following question: “Do you think the amount of sleep you have slept in the past 7 days is sufficient to recover from fatigue?” Participants could choose between the following responses: completely sufficient, sufficient, moderate, not sufficient, or not sufficient at all. In this study, “complete sufficient” and “sufficient” were classified as “sufficient”, “moderate” as “normal,” and “not sufficient” and “not sufficient at all” as “insufficient.” The questions about whether using of contraceptives, and contraceptive methods was applied only to those who answered “I have had sex” for the “Have you ever had sex?” Whether using contraception was evaluated using the following question: “Did you use contraception to prevent pregnancy during sexual intercourse?” Participants could choose between the following responses: always, mostly, occasionally, or never used contraception. In this study, “never used contraception” was classified as “no” and the other responses as “yes.” The contraceptive method was asked to those who answered that they “always,” “mostly,” or “occasionally” used contraception. The participants were asked to choose one of five options (medicine, condoms, coitus, interruptus, or menstrual cycle) to answer for the questions: “Which method of contraception is most commonly used during intercourse?”

For mental health characteristics, subjective health status, happiness, loneliness, sadness and despair, stress level, suicidal ideation, planning of suicide, and suicide attempts were used as variables. Subjective health status was evaluated using the following question: “How do you think your health is usually?” Participants could choose between the following responses: very healthy, healthy, normal, unhealthy, or very unhealthy. In this study, “very healthy” and “healthy” were regarded as “healthy,” “normal” as “normal” and “unhealthy” and “very unhealthy” as “unhealthy.” Happiness was evaluated using the following question: “How happy do you usually think?” Participants could choose between the following responses: very happy, slightly happy, normal, slightly unhappy, or very unhappy. In this study, “very happy” and “slightly happy” were regarded as “happy,” “normal” as “normal” and “slightly unhappy” and “very unhappy” as “unhappy.” Loneliness was evaluated using the following question: “In the last 12 months, how often have you felt lonely?” Participants could choose between the following responses: I didn't feel lonely at all, I almost never felt lonely, I sometimes felt lonely, I often felt lonely, or I always felt lonely. In this study, “I didn't feel lonely at all” and “I almost never felt lonely” were regarded as “hardly feel,” “I sometimes felt lonely” as “sometimes feel,” “I often felt lonely” and “I always felt lonely” were regarded as “often feel.” Sadness and despair were a response to “yes” or “no” to “In the past 12 months, have you ever felt so sad or despairing that you stopped your daily life for 2 weeks?” The stress level was evaluated using the following question: “How much stress do you usually feel?” Participants could choose between the following responses: feeling very much, feeling a lot, feeling a little, not feeling very much, or not feeling at all. In this study, “feeling very much” and “feeling a lot” were regarded as “often feel”, “feeling a little” as “sometimes feel,” “not feeling very much” and “not feeling at all” were regarded as “hardly feel.” In addition, the participants were asked to choose “yes” or “no” to answer for the questions: “During the past 12 months, have you thought, planned, or attempted suicide?”

### Data Analysis

The data collected in this study were analyzed using the IBM SPSS Statistics for Windows, Version 23.0. Armonk, NY: IBM Corp. The data provided by the 2020 KYRBS were collected by the method of complex sample design. The population structure, response rate, and weight of the population were reflected in this data according to the guidelines for complex sample analysis of the Korea Centers for Disease Control and Prevention.

The sexual intercourse item used in this study is a norminal variable divided into two groups (experienced and inexperienced). Chi-square test and logistic regression analysis is a method of estimating the relationship between dependent and independent variable on sexual intercourse frequency amongst Korean adolescents. In this study, demographic characteristics were input into Model 1, and health behavioral variables were input into Model 2. For Model 3, mental health variables were used based on our conceptual framework. Changes in related factors were confirmed in detail through the variables assigned to each model. This approach was used to overcome limitations of the multiple regression model, which only included independent variables to verify the influencing factors.

### Ethical Approval

This survey was a government-approved statistical survey (Approval No. 117058), and the researcher received approval from the institution for permission to use raw data from the website of the Korea Centers for Disease Control and Prevention. The Institutional Review Board of Jeonbuk National University (NO.: JBNU 2021-06-009) approved the deliberation exemption.

## Results

### Demographic Characteristics

The relationship between sexual intercourse according to the demographic characteristics of the participants is as follows (Table 1). In this study, all items of demographic characteristics showed a statistically significant difference regarding the presence or absence of sexual intercourse. Regarding the demographic characteristics of the participants, sexual intercourse was the highest among students in the third grade of middle school and high school, with 2.8 and 10.6%, respectively; in terms of gender, males in middle school and high school had engaged in sexual intercourse 2.4 and 9.3% more, respectively, then females. In terms of academic performance, sexual intercourse was the highest—at 2.5 and 9.2% in middle school and high school students, respectively—when their academic performances were “poor.” In terms of economic status, sexual intercourse was the highest among those with “low” economic status, at 2.6 and 10.1% for middle school and high school students, respectively. In terms of housing type, sexual intercourse was most common at 19.0 and 35.2% for middle school and high school students, respectively, when they were living in a “Orphanage”.

**Table 1:** Demographic characteristics of the participants (*n* = 54,948).

**Characteristic**	**Category**	**Sexual intercourse of middle school students**				**Sexual intercourse of high school students**			
		**Total *n* (%)**	**No *n* (%)**	**Yes *n* (%)**	**χ^2^ (*p*)**	**Total *n* (%)**	**No *n* (%)**	**Yes *n* (%)**	**χ^2^ (*p*)**
**Year level**	1st	10,005 (34.6)	9,879 (98.7)	126 (1.3)	62.85 (<0.001)	8,907 (34.3)	8,510 (95.5)	397 (4.5)	231.64 (<0.001)
	2nd	9,564 (33.0)	9,398 (98.3)	166 (1.7)		8,907 (34.3)	8,235 (92.5)	672 (7.5)	
	3rd	9,392 (32.4)	9,130 (97.2)	262 (2.8)		8,173 (31.5)	7,309 (89.4)	864 (10.6)	
**Gender**	Male	14,830 (51.2)	14,476 (97.6)	354 (2.4)	27.29 (<0.001)	13,523 (52.0)	12,261 (90.7)	1,262 (9.3)	98.96 (<0.001)
	Female	14,131 (48.8)	13,931 (98.6)	200 (1.4)		12,464 (48.0)	11,793 (94.6)	671 (5.4)	
**Academic performance**	Good	12,416 (42.9)	12,214 (98.4)	202 (1.6)	18.53 (<0.001)	7,730 (29.8)	7,230 (93.5)	500 (6.5)	56.17 (<0.001)
	Fair	8,438 (29.1)	8,292 (98.3)	146 (1.7)		8,147 (31.4)	7,642 (93.8)	505 (6.2)	
	Poor	8,107 (28.0)	7,901 (97.5)	206 (2.5)		10,110 (38.9)	9,182 (90.8)	928 (9.2)	
**Economic status**	Good	12,638 (43.6)	12,366 (97.9)	272 (2.2)	23.22 (<0.001)	8,701 (33.5)	7,972 (91.6)	729 (8.4)	75.87 (<0.001)
	Fair	13,295 (45.9)	13,092 (98.5)	203 (1.5)		13,102 (50.4)	12,322 (94.1)	780 (6.0)	
	low	3,028 (10.5)	2,949 (97.4)	79 (2.6)		4,184 (16.1)	3,760 (89.9)	424 (10.1)	
**Housing type**	With family	28,410 (98.1)	27,894 (98.2)	516 (1.8)	198.74 (<0.001)	23,922 (92.1)	22,196 (92.8)	1,726 (7.2)	219.59 (<0.001)
	With relative	134 (0.5)	124 (92.5)	10 (7.5)		130 (0.5)	110 (84.6)	20 (15.4)	
	Boarding house	44 (0.2)	37 (84.1)	7 (15.9)		198 (0.8)	144 (72.7)	54 (27.3)	
	Dormitory	294 (1.0)	288 (98.0)	6 (2.0)		1,632 (6.3)	1,536 (94.1)	96 (5.9)	
	Orphanage	79 (0.3)	64 (81.0)	15 (19.0)		105 (0.4)	68 (64.8)	37 (35.2)	

### Health Behavior Characteristics

The relationship between the presence of sexual intercourse according to the characteristics of the participant's health behavior is as follows (Table 2). Regarding the health behavior characteristics of middle school and high school students, alcohol consumption, smoking, and drug use were found to be higher in sexually ‘experienced’ than ‘not experience’ subjects. Sexual intercourse experience was more common among subjects who reported a lack of sleep, constituting 2.4 and 8.8% of middle school and high school students, respectively. With regard to students who reported having no sex education, 2.2% of middle school students had sexual intercourse, while 7.6% of high school students engaged in sexual intercourse. However, a significant statistical difference could only be confirmed in middle school students.

**Table 2:** Health behavior characteristics of the participants (*n* = 54,948).

**Characteristic**	**Category**	**Sexual intercourse of middle school students**				**Sexual intercourse of high school students**			
		**Total *n* (%)**	**No *n* (%)**	**Yes *n* (%)**	**χ^2^ (*p*)**	**Total *n* (%)**	**No *n* (%)**	**Yes *n* (%)**	**χ^2^ (*p*)**
**Drinking experience**	No	22,401 (77.4)	22,171 (99.0)	230 (1.0)	299.94 (<0.001)	14,190 (54.6)	13,867 (97.7)	323 (2.3)	1,186.91 (<0.001)
	Yes	6,560 (22.7)	6,236 (95.1)	324 (4.9)		11,797 (45.4)	10,187 (86.4)	1,610 (13.7)	
**Smoking experience**	No	27,395 (94.6)	27,045 (98.7)	350 (1.3)	784.16 (<0.001)	21,923 (84.4)	21,083 (96.2)	840 (3.8)	3,006.77 (<0.001)
	Yes	1,566 (5.4)	1,362 (87.0)	204 (13.0)		4,064 (15.6)	2,971 (73.1)	1,093 (26.9)	
**Sleep state**	Sufficient	10,353 (35.8)	10,169 (98.2)	184 (1.8)	13.05 (0.002)	6,471 (24.9)	6,069 (93.8)	402 (6.2)	43.21 (<0.001)
	Normal	9,936 (34.3)	9,774 (98.4)	162 (1.6)		8,720 (33.6)	8,141 (93.4)	579 (6.6)	
	Insufficient	8,672 (29.9)	8,464 (97.6)	208 (2.4)		10,796 (41.5)	9,844 (91.2)	952 (8.8)	
**Drug use**	No	28,795 (99.4)	28,260 (98.1)	535 (1.9)	73.73 (<0.001)	25,748 (99.1)	23,878 (92.7)	1,870 (7.3)	102.83 (<0.001)
	Yes	166 (0.6)	147 (88.6)	19 (11.5)		239 (0.9)	176 (73.6)	63 (26.4)	
**Sex education experience**	No	6,975 (24.1)	6,823 (97.8)	152 (2.2)	5.89 (0.015)	8,748 (33.7)	8,124 (92.9)	624 (7.1)	0.05 (0.827)
	Yes	21,986 (75.9)	21,584 (98.2)	402 (1.8)		17,239 (66.3)	15,930 (92.4)	1,309 (7.6)	
**Contraception**	No	229 (41.3)	0 (0)	229 (41.3)	–	358 (18.5)	0 (0)	358 (18.5)	–
	Yes	325 (58.7)	0 (0)	325 (58.7)		1,575 (81.5)	0 (0)	1,575 (81.5)	
**Contraceptive method**	Medicine	32 (9.9)	0 (0)	32 (9.9)	–	46 (2.9)	0 (0)	46 (2.9)	–
	Condoms	243 (74.8)	0 (0)	243 (74.8)		1,332 (84.6)	0 (0)	1,332 (84.6)	
	Coitus interruptus and menstrual cycle	50 (15.4)	0 (0)	50 (15.4)		197 (12.5)	0 (0)	197 (12.5)	

### Mental Health Characteristics

The relationship between sexual intercourse and the participant's mental health characteristics is as follows (Table 3). Regarding the mental health characteristics of the participants, sexual intercourse was the highest among middle school and high school students −2.6 and 9.3%, respectively— who perceived themselves as “unhealthy,” and there was a statistically significant difference only in high school students. Regarding the subjective state of happiness, subjective loneliness, the level of stress, sadness and despair, the suicidal ideation, suicide plan, and suicide attempt, there were statistically significant differences in both middle and high school students.

**Table 3:** Mental health characteristics of the participants (*n* = 54,948).

**Characteristic**	**Category**	**Sexual intercourse of middle school students**				**Sexual intercourse of high school students**			
		**Total *n* (%)**	**No *n* (%)**	**Yes *n* (%)**	**χ^2^ (*p*)**	**Total *n* (%)**	**No *n* (%)**	**Yes *n* (%)**	**χ^2^ (*p*)**
**Subjective health status**	Healthy	20,764 (71.7)	20,375 (98.1)	389 (1.9)	1.91 (0.385)	17,680 (68.0)	16,356 (92.5)	1,324 (7.5)	15.43 (<0.001)
	Normal	6,441 (22.2)	6,321 (98.1)	120 (1.9)		5,901 (22.7)	5,515 (93.5)	386 (6.5)	
	Unhealthy	1,756 (6.1)	1,711 (97.4)	45 (2.6)		2,406 (9.3)	2,183 (90.7)	223 (9.3)	
**Subjective state of happiness**	Happy	19,336 (66.8)	19,005 (98.3)	331 (1.7)	46.12 (<0.001)	15,839 (61.0)	14,770 (93.3)	1,069 (6.8)	84.22 (<0.001)
	Normal	7,350 (25.4)	7,217 (98.2)	133 (1.8)		7,610 (29.3)	7,065 (92.8)	545 (7.2)	
	Unhappy	2,275 (7.9)	2,185 (96.0)	90 (4.0)		2,538 (9.8)	2,219 (87.4)	319 (12.6)	
**Subjective loneliness**	Hardly feel	15,670 (54.1)	15,434 (98.5)	236 (1.5)	86.45 (<0.001)	12,589 (48.4)	11,872 (94.3)	717 (5.7)	179.47 (<0.001)
	Sometimes feel	9,541 (32.9)	9,376 (98.3)	165 (1.7)		9,382 (36.1)	8,685 (92.6)	697 (7.4)	
	Often feel	3,750 (13.0)	3,597 (95.9)	153 (4.1)		4,016 (15.5)	3,497 (87.1)	519 (12.9)	
**Stress level**	Often feel	8,899 (30.7)	8,651 (97.2)	248 (2.8)	33.07 (<0.001)	9,763 (37.6)	8,873 (90.9)	890 (9.1)	47.84 (<0.001)
	Sometimes feel	13,058 (45.1)	12,862 (98.5)	196 (1.5)		11,321 (43.6)	10,611 (93.7)	710 (6.3)	
	Hardly feel	7,004 (24.2)	6,894 (98.4)	110 (1.6)		4,903 (18.9)	4,570 (93.2)	333 (6.8)	
**Sadness and despair**	No	22,221 (76.7)	21,908 (98.6)	313 (1.4)	76.94 (<0.001)	18,887 (72.7)	17,803 (94.3)	1,084 (5.7)	242.96 (<0.001)
	Yes	6,740 (23.3)	6,499 (96.4)	241 (3.6)		7,100 (27.3)	6,251 (88.0)	849 (12.0)	
**Suicidal ideation**	No	25,948 (89.6)	25,536 (98.4)	412 (1.6)	104.72 (<0.001)	23,021 (88.6)	21,514 (93.5)	1,507 (6.6)	194.75 (<0.001)
	Yes	3,013 (10.4)	2,871 (95.3)	142 (4.7)		2,966 (11.4)	2,540 (85.6)	426 (14.4)	
**Suicide plan**	No	27,900 (96.3)	27,413 (98.3)	487 (1.8)	89.39 (<0.001)	25,095 (96.6)	23,344 (93.0)	1,751 (7.0)	221.71 (<0.001)
	Yes	1,061 (3.7)	994 (93.7)	67 (6.3)		892 (3.4)	710 (79.6)	182 (20.4)	
**Suicide attempt**	No	28,369 (98.0)	27,871 (98.2)	498 (1.8)	169.26 (<0.001)	25,458 (98.0)	23,676 (93.0)	1,782 (7.0)	350.39 (<0.001)
	Yes	592 (2.0)	536 (90.5)	56 (9.5)		529 (2.0)	378 (71.5)	151 (28.5)	

### Factors Associated With Sexual Intercourse

Logistic regression analysis identified factors associated with sexual intercourse among middle and high school students as follows (Tables 4, 5). Simple logistic regression analysis was performed to calculate the crude odds ratio (OR) for each variable. The factors related to sexual intercourse were analyzed by multiple logistic regression models. This study presented three models. Demographics were used for Model 1, and Model 2 considered both health behaviors and demographics. Model 3 added mental health characteristics to demographics and health behaviors.

**Table 4:** Factors affecting sexual intercourse in middle school students (*n* = 28,961).

**Level**	**Characteristic**	**Category**	**Crude**		**Model 1^a^**		**Model 2^b^**		**Model 3^c^**	
			**OR (95% CI)**	***p*** **-Value**	**OR (95% CI)**	***p*** **-Value**	**OR (95% CI)**	***p*** **-Value**	**OR (95% CI)**	***p*** **-Value**
**Demographic characteristics**	Year level	2nd	1.340 (1.0291.745)	0.030	1.245 (0.9551.623)	0.105	1.064 (0.8071.405)	0.659	1.093 (0.828–1.441)	0.531
		3rd	2.219 (1.758–2.802)	<0.001	2.067 (1.6362.611)	<0.001	1.380 (1.0881.752)	0.008	1.457 (1.148–1.848)	0.002
		1st	ref		ref		ref		ref	
	Gender	Male	1.671 (1.368–2.041)	<0.001	1.636 (1.338–2.001)	<0.001	1.391 (1.1351.704)	0.002	1.632 (1.317–2.022)	<0.001
		Female	ref		ref		ref		ref	
	Academic performance	Fair	1.068 (0.8271.380)	0.612	1.192 (0.918–1.549)	0.188	1.117 (0.854–1.461)	0.418	1.122 (0.854–1.473)	0.410
		Poor	1.611 (1.270–2.043)	<0.001	1.582 (1.231–2.032)	<0.001	1.090 (0.843–1.411)	0.511	1.053 (0.811–1.367)	0.698
		Good	ref		ref		ref		ref	
	Economic status	Fair	0.665 (0.540–0.817)	<0.001	0.601 (0.486–0.744)	<0.001	0.632 (0.511–0.782)	<0.001	0.645 (0.522–0.797)	<0.001
		Low	1.246 (0.943–1.647)	0.122	0.955 (0.713–1.279)	0.758	0.815 (0.600–1.109)	0.194	0.705 (0.516–0.963)	0.028
		Good	ref		ref		ref		ref	
	Housing type	With relative	3.649 (1.692–7.866)	<0.001	3.616 (1.631–8.016)	0.002	2.954 (1.220–7.150)	0.016	2.616 (1.146–5.972)	0.022
		Boarding house	8.735 (3.283–23.239)	<0.001	7.499 (2.785–20.192)	<0.001	3.541 (0.993–12.630)	0.051	3.305 (0.83813.042)	0.088
		Dormitory	1.944 (0.807–4.682)	0.138	1.658 (0.696–3.946)	0.254	1.629 (0.597–4.446)	0.340	1.652 (0.626–4.358)	0.311
		Orphanage	17.596 (8.756–35.363)	<0.001	13.802 (6.523–29.203)	<0.001	12.591 (5.807–27.303)	<0.001	10.698 (4.99322.918)	<0.001
		With family	ref		ref		ref		ref	
**Health behavior characteristics**	Drinking experience	Yes	4.641 (3.826–5.630)	<0.001			2.457 (1.960–3.079)	<0.001	2.229 (1.774–2.801)	<0.001
		No	ref				ref		ref	
	Smoking experience	Yes	11.327 (9.177–13.980)	<0.001			5.772 (4.536–7.344)	<0.001	5.165 (4.047–6.591)	<0.001
		No	ref				ref		ref	
	Sleep state	Normal	0.855 (0.663–1.101)	0.224			0.725 (0.561–0.938)	0.015	0.716 (0.550–0.933)	0.013
		Insufficient	1.299 (1.031–1.638)	0.027			0.994 (0.788–1.254)	0.960	0.858 (0.670–1.098)	0.223
		Sufficient	ref				ref		ref	
	Drug use	Yes	6.936 (4.119–11.681)	<0.001			2.613 (1.452–4.703)	0.001	1.692 (0.928–3.088)	0.086
		No	ref				ref		ref	
	Sex education experience	Yes	0.774 (0.628–0.954)	0.016			0.823 (0.664–1.021)	0.076	0.815 (0.659–1.008)	0.060
		No	ref				ref		ref	
**Mental health characteristics**	Subjective health status	Normal	1.001 (0.793–1.262)	0.995					0.964 (0.759–1.223)	0.761
		Unhealthy	1.281 (0.901–1.821)	0.167					0.793 (0.542–1.160)	0.232
		Healthy	ref						ref	
	Subjective state of happiness	Normal	0.968 (0.782–1.198)	0.766					0.772 (0.617–0.966)	0.024
		Unhappy	2.284 (1.753–2.976)	<0.001					0.939 (0.642–1.372)	0.744
		Happy	ref						ref	
	Subjective loneliness	Often feel	1.016 (0.800–1.290)	0.895					0.919 (0.705–1.199)	0.535
		Sometimes feel	2.673 (2.136–3.346)	<0.001					1.501 (1.062–2.121)	0.022
		Hardly feel	ref						ref	
	Stress level	Often feel	1.585 (1.184–2.123)	0.002					1.029 (0.715–1.480)	0.879
		Sometimes feel	0.814 (0.608–1.090)	0.167					0.808 (0.597–1.093)	0.166
		Hardly feel	ref						ref	
	Sadness and despair	Yes	2.420 (1.976–2.963)	<0.001					1.493 (1.126–1.980)	0.005
		No	ref	<0.001					ref	
	Suicidal ideation	Yes	2.915 (2.352–3.611)						1.152 (0.794–1.671)	0.457
		No	ref						ref	
	Suicide plan	Yes	3.824 (2.833–5.163)	<0.001					1.142 (0.689–1.895)	0.606
		No	ref						ref	
	Suicide attempt	Yes	6.116 (4.475–8.358)	<0.001					1.920 (1.208–3.051)	0.006
		No	ref						ref	

a *Model 1: Nagelkerke R^2^ was 0.269 (Cox and Snell R^2^ was 0.268)*.

b *Model 2: Nagelkerke R^2^ was 0.649 (Cox and Snell R^2^ was 0.648)*.

c *Model 3: Nagelkerke R^2^ was 0.695 (Cox and Snell R^2^ was 0.695)*.

*OR: odds ratio; CI: confidence interval*.

**Table 5:** Factors affecting sexual intercourse in high school students (*n* = 25,987).

**Level**	**Characteristic**	**Category**	**Crude**		**Model 1^a^**		**Model 2^b^**		**Model 3^c^**	
			**OR (95% CI)**	***p*** **-Value**	**OR (95% CI)**	***p*** **-Value**	**OR (95% CI)**	***p*** **-Value**	**OR (95% CI)**	***p*** **-Value**
**Demographic characteristics**	Year level	2nd	1.749 (1.506–2.032)	<0.001	1.741 (1.499–2.022)	<0.001	1.572 (1.349–1.833)	<0.001	1.563 (1.340–1.823)	<0.001
		3rd	2.459 (2.105–2.874)	<0.001	2.462 (2.112–2.870)	<0.001	1.962 (1.679–2.293)	<0.001	1.989 (1.703–2.324)	<0.001
		1st	ref		ref		ref		ref	
	Gender	Male	1.865 (1.628–2.136)	<0.001	1.822 (1.594–2.082)	<0.001	1.266 (1.106–1.449)	0.001	1.429 (1.237–1.649)	<0.001
		Female	ref		ref		ref		ref	
	Academic performance	Fair	0.925 (0.801–1.069)	0.290	1.047 (0.903–1.212)	0.545	1.011 (0.866–1.180)	0.892	1.012 (0.867–1.181)	0.880
		Poor	1.417 (1.254–1.602)	<0.001	1.582 (1.383–1.810)	<0.001	1.177 (1.016–1.363)	0.030	1.165 (1.003–1.354)	0.045
		Good	ref		ref		ref		ref	
	Economic status	Fair	0.689 (0.610–0.778)	<0.001	0.659 (0.581–0.747)	<0.001	0.706 (0.620–0.805)	<0.001	0.727 (0.638–0.830)	<0.001
		low	1.228 (1.057–1.427)	0.008	1.021 (0.871–1.195)	0.801	0.988 (0.833–1.173)	0.893	0.973 (0.819–1.156)	0.755
		Good	ref		ref		ref		ref	
	Housing type	With relative	2.149 (1.255–3.679)	0.005	2.087 (1.204–3.616)	0.009	1.972 (1.030–3.774)	0.040	1.919 (1.012–3.639)	0.046
		Boarding house	5.221 (3.630–7.508)	<0.001	4.745 (3.331–6.760)	<0.001	3.930 (2.623–5.887)	<0.001	3.661 (2.484–5.396)	<0.001
		Dormitory	0.847 (0.654–1.096)	0.205	0.864 (0.660–1.132)	0.289	0.901 (0.688–1.180)	0.449	0.884 (0.668–1.171)	0.391
		Orphanage	7.618 (4.725–12.282)	<0.001	6.516 (3.803–11.162)	<0.001	4.723 (2.599–8.585)	<0.001	3.566 (1.863–6.824)	<0.001
		With family	ref		ref		ref		ref	
**Health behavior characteristics**	Drinking experience	Yes	6.493 (5.638–7.478)	<0.001			2.981 (2.562–3.467)	<0.001	2.840 (2.434–3.315)	<0.001
		No	ref				ref		ref	
	Smoking experience	Yes	9.239 (8.167–10.453)	<0.001			5.095 (4.436–5.853)	<0.001	4.746 (4.141–5.439)	<0.001
		No	ref				ref		ref	
	Sleep state	Normal	1.090 (0.947–1.256)	0.230			0.999 (0.862–1.159)	0.994	1.002 (0.859–1.169)	0.978
		Insufficient	1.470 (1.286–1.682)	<0.001			1.224 (1.059–1.415)	0.006	1.151 (0.988–1.341)	0.070
		Sufficient	ref				ref		ref	
	Drug use	Yes	4.442 (3.233–6.104)	<0.001			3.218 (2.142–4.836)	<0.001	2.511 (1.635–3.857)	<0.001
		No	ref				ref		ref	
	Sex education experience	Yes	1.013 (0.903–1.136)	0.827			1.065 (0.944–1.202)	0.305	1.054 (0.933–1.190)	0.400
		No	ref				ref		ref	
**Mental health characteristics**	Subjective health status	Normal	0.849 (0.751–0.960)	0.009					0.814 (0.711–0.933)	0.003
		Unhealthy	1.245 (1.045–1.482)	0.014					0.896 (0.718–1.117)	0.329
		Healthy	ref						ref	
	Subjective state of happiness	Normal	1.016 (0.911–1.133)	0.776					0.865 (0.755–0.991)	0.037
		Unhappy	1.897 (1.635–2.200)	<0.001					0.841 (0.669–1.058)	0.139
		Happy	ref						ref	
	Subjective loneliness	Often feel	1.273 (1.135–1.429)	<0.001					1.018 (0.884–1.173)	0.801
		Sometimes feel	2.366 (2.095–2.671)	<0.001					1.270 (1.049–1.538)	0.014
		Hardly feel	ref						ref	
	Stress level	Often feel	1.338 (1.165–1.537)	<0.001					0.943 (0.785–1.132)	0.530
		Sometimes feel	0.921 (0.795–1.067)	0.274					0.930 (0.794–1.088)	0.364
		Hardly feel	ref						ref	
	Sadness and despair	Yes	2.219 (2.011–2.450)	<0.001					1.506 (1.311–1.730)	<0.001
		No	ref						ref	
	Suicidal ideation	Yes	2.402 (2.125–2.716)	<0.001					1.113 (0.912–1.359)	0.292
		No	ref						ref	
	Suicide plan	Yes	3.622 (3.028–4.332)	<0.001					1.402 (1.047–1.880)	0.024
		No	ref						ref	
	Suicide attempt	Yes	5.354 (4.402–6.512)	<0.001					2.071 (1.509–2.841)	<0.001
		No	ref						ref	

a *Model 1: Nagelkerke R^2^ was 0.730 (Cox and Snell R^2^ was 0.730)*.

b *Model 2: Nagelkerke R^2^ was 0.993 (Cox and Snell R^2^ was 0.993)*.

c *Model 3: Nagelkerke R^2^ was 0.995 (Cox and Snell R^2^ was 0.995)*.

*OR: odds ratio; CI: confidence interval*.

In the final model 3 demographic characteristics of middle school students, the most influential related factor for sexual intercourse was ‘orphanage’, which was 10.698 times [95% confidence interval (CI): 4.993–22.918] higher than that of living with family. In high school students, the most influential factor related to sexual intercourse was ‘bording house’, which was 3.661 times (95% CI: 2.484–5.396) higher than that of living with family. In middle school students, it was statistically significant in all items of demographic characteristics, but in high school students, it was not statistically significant in ‘Academic performance’.

In health behavioral characteristics, “smoking experience” was the most influential related factor for both middle school and high school students. It was 5.165 times (95% CI: 4.047–6.591) and 4.746 times (95% CI: 4.141–5.439) higher than “No,” respectively. The “sex education experience” was not statistically significant for both middle school and high school students.

Finally, in terms of mental health characteristics, the most influential factor related to sexual intercourse was “suicide attempt” in both middle and high school students. It was 1.920 times (95% CI: 1.208–3.051) and 2.071 times (95% CI: 1.509–2.841) higher than “No,” respectively. Both “stress level” and “suicidal ideation” were not statistically significant in middle school and high school students.

## Discussion

This study used data from the KYRBS, which provided national youth health statistics, to analyze the factors associated with sexual intercourse in adolescents. It was found that year level, gender, housing type, drinking experience, smoking experience, loneliness, sadness, despair, suicide plans, and suicide attempts were associated with sexual intercourse in both middle school and high school students.

Comparing sexual experiences by gender, male students had a higher rate of sexual intercourse than females. This was consistent with studies among adolescents from the United States (21), Indonesia (17), and Thailand (22). In relation to sexual intercourse, male students were more likely to smoke, drink, and use drugs than female students and previous studies that reported males' unhealthy lifestyles compared to female students related to sexual intercourse (17, 21, 22). However, in this study, we did not analyze gender differences in alcohol consumption, smoking rates, and drug use. Therefore, further research is required.

In the health behaviors, alcohol consumption and smoking were correlated to sexual experience in both middle school and high school students, while drug experience affected sexual intercourse frequency only in high school students. This is consistent with previous studies illustrating a positive correlation between drinking (13, 22, 23), smoking (13, 24), and drug use (23) with sexual intercourse. In a study examining the occurrence of drug and alcohol use in sexually-active American adolescents, 8 out of 10 reported using both marijuana and alcohol (23). Of these, about two-thirds simultaneously engaged in sexual intercourse and marijuana use. Similarly, more than half simultaneously engaged in sex and alcohol use, and half reported performing all three actions on the same day (23). Additionally, according to the 2015 Youth Risk Behavior Surveillance (YRBS) (25), 20.6% of high school students who have had sexual intercourse reported that they recently drank alcohol or used drugs prior to sexual intercourse (15). Alcohol myopia theory posits that alcohol consumption promotes sexual activity through behavioral inhibition caused by information processing impairment (26, 27). Alcohol consumption or the use of substance can cause severe cognitive decline, leading to impulsive actions such as indiscreet sexual behavior, sexual assault, and the use of additional psychostimulants (28, 29). Additionally, the use of substances acts as a gateway for sexual intercourse (30). For this reason, it appears that sexual experiences are higher among students who drink, smoke, and take drugs.

Additionally, many studies have shown that drinking, smoking, and drug use are risk factors for unprotected sexual intercourse (31). Furthermore, when alcohol was consumed, adolescents engaged in more sexual intercourse and were more likely to not use condoms (13). Among study adolescents who had sexual intercourse, 41.3% did so without contraception which is a high rate compared to 13.8% surveyed in the YRBS in the United States (21). This indicates that Korean adolescents are exposed to a high risk of sexually transmitted infections or premature pregnancy. As well, complications during pregnancy and birth are more common in adolescents than in adult women, as adolescents are not completely developed physically, including incomplete pelvic development (32).

Adolescent mothers are placed in a difficult social and economic situation (33) as their education is often interrupted leading to difficulty finding employment (34, 35). In addition, physical and emotional changes resulting from pregnancy and child birth endured at a young age can cause burdens in the future (33).

This suggests that sex education—combined with a preventive approach to alcohol intake and smoking— and programs for contraception and the prevention of sexually transmitted diseases are needed.

Furthermore, in this study, sexual intercourse showed a significant positive correlation with suicide attempts in middle school students and suicide plans in high school students. These findings lend support to previous researches that had shown a positive association between suicidal ideation and sexual activity (2, 17). In a study of adolescents in 38 countries (6), it was reported that suicide attempts and sexual relationships were related, and the same results were reported in a study of Baiden et al. (36). The current study showed that sexual intercourse among adolescents was associated with problematic behaviors—such as drinking, smoking, and drug use—and psychological behaviors—such as loneliness, sadness, despair, suicidal ideation, and suicidal attempts—that could occur together (2, 36). Furthermore, a Swedish study found that girls who experienced sexual intercourse at an early age had poor physical and mental health (2). Bandura's concept of self-efficacy suggests that depressed individuals have decreased capability to control their lives, including areas related to sexual behavior (2, 17, 37). Our findings are consistent with this theory, as both middle and high school students who had engaged in sexual intercourse reported increased levels of loneliness, sadness and despair, and suicide attempts. Therefore, when creating sexual education programs, it is essential to incorporate an understanding of adolescents' mental health concerns.

Sexual behavior can be influenced by many physiological factors, as well as rapidly changing cultural and social pressures; understanding these influencing factors can provide intervention regarding information that can help adolescents make appropriate sexual behavior choices, as well as help protect their rights related to these choices. Additionally, promoting appropriate attitudes toward sexual behavior in adolescence can help adolescents understand contraception and may lead to a decrease in the rate of unintended pregnancies under the age of 18 (31). Therefore, this study is important since it uses data from the KYRBS to identify sexual experiences of middle school and high school adolescents, provide basic data for future youth sexual health intervention programs, and provide a basis for laying the foundation for sex education-related policies.

### Limitations of the Study and Implications for Research and Practice

There are some limitations to this study. The KYRBS does not include adolescents not in school, thus, the results of this study cannot be generalized among all adolescents in Korea. To supplement this, further studies on out-of-school youth are proposed. Additionally, there is a limit to the accuracy of the survey given the subjective nature of the questions regarding online and sensitive topics. Therefore, further research with qualitative evaluation is required to supplement the reliability of research data.

## Conclusion

This was a secondary data analysis study to identify factors associated with sexual intercourse among Korean adolescents based on data from the 2020 KYRBS. This research demonstrates that the relationships between sexual intercourse experience, and variables including demographics, health behaviors, and mental health, differ according to school level. There were differences in related factors according to the school level. The results of this study may be used as basic data for developing and counseling intervention programs regarding the promotion of proper sexual health practices through early screening of negative factors related to sexual intercourse experience in terms of health type and mental health. In future research, it would be advantageous for variables used in this study to have further detail and quantification. This will accurately express the properties of the sample and ensure the legitimacy of the study.

## Data Availability Statement

The original contributions presented in the study are included in the article/supplementary material, further inquiries can be directed to the corresponding author.

## Ethics Statement

The Institutional Review Board of Jeonbuk National University (NO.: JBNU 2021-06-009) approved the deliberation exemption. Written informed consent from the participants' legal guardian/next of kin was not required to participate in this study in accordance with the national legislation and the institutional requirements.

## Author Contributions

Conceptualization, writing—original draft preparation, and writing—review and editing: JB and JS. Methodology, investigation, and data curation: SH. All authors have read and agree to the published version of the manuscript.

## Conflict of Interest

The authors declare that the research was conducted in the absence of any commercial or financial relationships that could be construed as a potential conflict of interest.

## Publisher's Note

All claims expressed in this article are solely those of the authors and do not necessarily represent those of their affiliated organizations, or those of the publisher, the editors and the reviewers. Any product that may be evaluated in this article, or claim that may be made by its manufacturer, is not guaranteed or endorsed by the publisher.
